# Human biomonitoring of heavy metals in the vicinity of non-ferrous metal plants in Ath, Belgium

**DOI:** 10.1186/s13690-016-0154-8

**Published:** 2016-10-03

**Authors:** Sébastien Fierens, Javiera Rebolledo, Ann Versporten, Ethel Brits, Vincent Haufroid, Pierre De Plaen, An Van Nieuwenhuyse

**Affiliations:** 1 Direction of Public Health and Surveillance, Health and Environment Service, Scientific Institute of Public Health (WIV-ISP), Brussels, Belgium; 2 Department of Clinical Biology, Laboratory of Industrial and Environmental Toxicology, University Hospital Saint-Luc, Université catholique de Louvain (UCL), Brussels, Belgium; 3 Department of Public Health, Section of Occupational, Environmental and Insurance Medicine, Katholieke Universiteit Leuven, Leuven, Belgium

**Keywords:** Human biomonitoring, Heavy metals, Lead, Cadmium, Biomarkers, Retinol-binding protein, Albumin

## Abstract

**Background:**

A previous study revealed an environmental contamination by heavy metals in the vicinity of two non-ferrous metal plants in Ath, Belgium. The purpose of the current cross-sectional study was to estimate exposure of the population to heavy metals in the vicinity of the plants, in comparison with population living further away.

**Methods:**

We did a random sampling in the general population of Ath in two areas: a central area, including the plants, and a peripheral area, presumably less exposed. We quantified cadmium, lead, nickel, chromium and cobalt in blood and/or urine of children and adults in three age groups: (i) children aged 2.5 to 6 years (*n* = 98), (ii) children aged 7 to 11 years (*n* = 74), and (iii) adults aged 40 to 60 years (*n* = 106). We also studied subclinical health effects by quantifying retinol-binding protein and microalbuminuria, and by means of a Strengths and Difficulties Questionnaire.

**Results:**

We obtained a participation rate of 24 %. Blood lead levels were significantly higher in young children living in the central area (18.2 μg/l ; 95 % CI: 15.9–20.9) compared to the peripheral area (14.8 μg/l ; 95 % CI: 12.6–17.4). We observed no other significant mean difference in metal concentrations between the two areas. In the whole population, blood lead levels were higher in men (31.7 μg/l ; 95 % CI: 27.9–36.1) than in women (21.4 μg/l ; 95 % CI: 18.1–25.3). Urine cadmium levels were 0.06 μg/g creatinine (95 % CI: 0.05–0.07), 0.21 μg/g creatinine (95 % CI: 0.17–0.27), and 0.25 μg/g creatinine (95 % CI: 0.20–0.30) for children, men, and women, respectively.

**Conclusions:**

Despite higher blood lead levels in young children living close to the plants, observed metal concentrations remain in the range found in other similar biomonitoring studies in the general population and are below the levels of concern for public health.

## Background

In industrialized countries, environmental pollution by heavy metals occurred *via* a wide range of processes and pathways. Lead emissions were mainly related to road transport while cadmium emissions were primarily associated with non-ferrous metallurgy and fuel combustion [1]. From the middle of the 19^th^ century, production of heavy metals increased steeply for more than 100 years, with concomitant emissions into the environment. At the end of the 20^th^ century, however, emissions of heavy metals started to decrease in high income countries [1]. Although emissions from industrial sites have declined overall, soils can remain contaminated for decades, which is still a concern for populations living in polluted areas. Furthermore, in the case of contaminated soils, pollutants can be captured by plants and therefore enter into the food chain, where bioconcentration of pollutants can occur, leading to an increased exposure of people eating contaminated foods.

The health effects associated with metal exposure are not only documented in populations having experienced high exposure levels such as in occupational conditions, but also at environmental exposure levels, in adults and children [2]. This is of particular concern given the greater sensitivity of children to heavy metals compared to adults. The target organs for toxicity of heavy metals vary according to the pollutant, and effects depend on the intensity and duration of exposure. Low levels of lead can, over time, damage the central nervous system, but also heart and kidneys [3, 4]. Young children are particularly sensitive to neuro-developmental toxicity of lead [5, 6]. Some studies suggest that the chronic low environmental exposure to cadmium can adversely affect the general population [7].

The presence of two still operating plants, from the non-ferrous metal industry, in the Belgian municipality of Ath, a residential area in Wallonia, has long been a concern for the local population and authorities. The first plant, located at about 400 m west from the city center, is established in Ath since the beginning of last century and is involved in the chemistry and metallurgy of non-ferrous metals (zinc, lead, nickel and cadmium salts). It is one of the major producers of cadmium salts in the world. The second plant, located at about 800 m from the city center and about 400 m away from the first plant, is specialized in manufacturing metal alloy powders. In 2006, the local authorities have undertaken an assessment of potential environmental pollution. This assessment revealed relatively high levels of heavy metals in surface soil samples collected near houses located in the vicinity of these plants [8]. The highest values (up to 50 ppm of cadmium and 275 ppm of lead) were observed within 500 m around the plants. Moreover, the Walloon monitoring network of air quality has recurrently reported high levels of metals in dust depositions in Ath. Values recorded at the measuring station of Ath were often the highest observed in Wallonia. For example, the median value of cadmium immission (deposition) was 23 μg/m^2^.d in 2004 [9].

We conducted the present study in order to evaluate the potential transfer of this environmental contamination into the human body and the consequences on health for the population of Ath, using human biomonitoring methods (HBM).

## Methods

### Study design

We performed in 2009 a population based cross-sectional study. Detailed information on methodological aspects of this study can be found in a paper previously published in this journal [10]. We used HBM to assess environmental exposure of the population to five metals: lead, cadmium, nickel, chromium, and cobalt, in the vicinity of the plants in comparison with population living further away. We also studied subclinical health effects by means of a Strengths and Difficulties Questionnaire, and by quantifying retinol-binding protein and microalbuminuria. Indeed, the first organ affected by cadmium and displaying signs of toxicity is the kidney, especially the proximal tubular cells, the main site of cadmium accumulation. The earliest manifestation of cadmium-induced renal damage is the increased urinary excretion of microproteins (molecular weight <40 kD). Retinol-binding protein (RBP) is one of such protein, and has been validated for the screening of tubular proteinuria [11]. At a more advanced stage, the glomerular filtration rate can be impaired. Albumin is one of the molecules excreted in greater amounts in case of cadmium nephropathy. The microalbuminuria (urinary albumin between 20 and 200 mg/l) is used as indicator of early glomerular damage [12].

We submitted this study to the Belgian Commission for the Protection of Privacy, and the study received the approval of the Ethics Commission of the Medicine Faculty of the *Université catholique de Louvain* (UCL) with the Belgian registration number B40320095714. Each participant has given its signed informed consent. For children, the informed consent was given by their parents or tutors.

### Study area

**Fig. 1:**
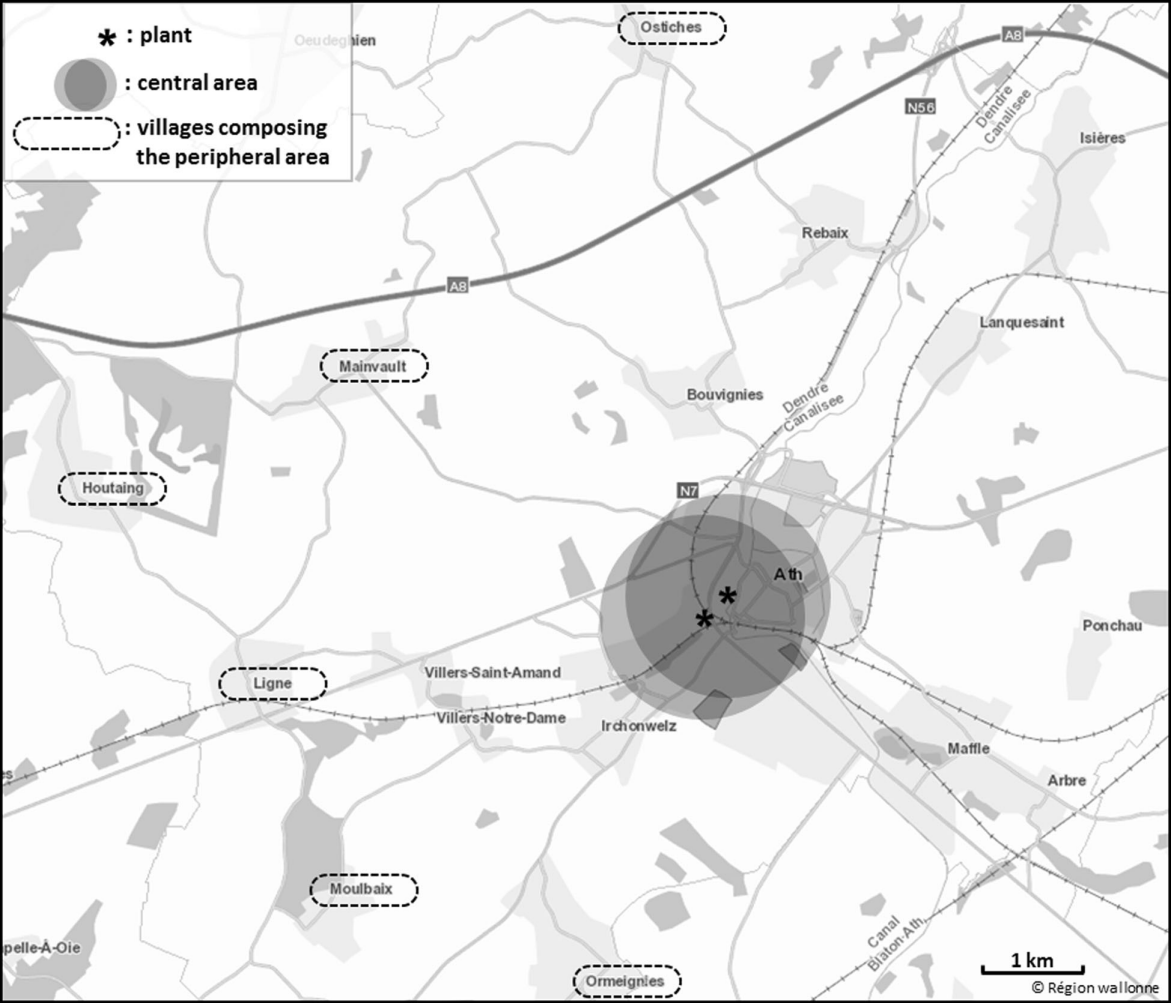
Map of the study area, Ath, Belgium, 2009

The study was carried out in the municipality of Ath, which belongs to the province of Hainaut, in the Walloon Region of Belgium. The municipality of Ath had 27,586 inhabitants in 2008, the year when the study protocol was created. Ath is located in a mainly agricultural area. The city is mostly residential but includes two non-ferrous metal plants, still in operation. We defined two areas of investigation. The first one, called the ‘central area’, was formed by a circle of 1 km radius around each of the two plants (Fig. 1). This radius was selected in order to target the most exposed area to metals emitted by the plants, based on extrapolation of metal particulate deposition and on environmental data (metal concentrations are maximal in soil samples within 500 m around the plants). Moreover, this radius of 1 km include the entire city center of Ath but exclude further areas where larger road network could lead to traffic pollution interfering as environmental source of exposure. We assumed that the central area included the most exposed population. The second area, called the ‘peripheral area’, was located further away from the plants. It included a population assumed to be little or not exposed to the metals emitted by the two plants. The peripheral area was composed of six villages within the municipality of Ath, located at more than three kilometers away from the plants (Fig. 1). These villages were selected because of their location outside the corridor of prevailing winds, blowing in the direction of the North-East, and because of the low likelihood of presence of other known sources of exposure such as dense traffic.

### Participants

We investigated three target groups: young children aged 2.5 to 6 years, children aged 7 to 11 years, and adults aged 40 to 60 years. The time of residence in the study area had to be at least 10 years for adults, 1 year for children aged 7 to 11 years, and 6 months for children aged 2.5 to 6 years. We investigated approximately equal proportions of both sexes. Because of substantial differences in metals body burden that could be observed between sexes, we recruited men and women independently and interpreted the results separately. We recruited participants at random amongst individuals fulfilling the criteria described above, stratified by the three target groups and with an equal sample size between the two studied areas.

We used population lists provided by the authorities of Ath, containing information allowing the selection of the right range of age, time of residence, and geographical area. We firstly sent an information document by mail to all the eligible population. A professional company of sample survey did subsequently the recruitment by phone. Subjects with occupational exposure were excluded in this step. For more details on the recruitment procedure, see the methodological article by Rebolledo et al. [10].

### Study size

We calculated the sample size in order to reach the level of significance for a difference in blood lead values of 30 % in comparison to representative values. The expected sample size was 284 persons in total for the study.

### Measurement

#### Biological samples

We took all samples in the same place in Ath, between 26^th^ February 2009 and 20^th^ March 2009. Adults and children aged 7 to 11 years provided a urine sample in a metal-free container (NOVOLAB) by the midstream technique. They also gave two tubes of venous blood, taken by a physician. The tubes were certified trace element free. We used the first one, containing EDTA (BD Vacutainer trace element plastic K2EDTA), for the measurement of lead and cadmium, and the second one, containing lithium and heparin (BD Vacutainer plasma tube plastic), for the measurement of serum ferritin. This protein, correlated with iron levels in the body, is used as determinant of anemia, which is known for increasing gastro-intestinal absorption of cadmium and lead [13]. We adopted a less invasive procedure for young children aged 2.5 to 6 years: we took approximately 10 droplets of capillary blood by a little prick at the top of the digit, using specific micro-tubes (BD Microtainer). This sample was intended to measure lead, the only biomarker quantified for this age group.

At the end of each sampling day, we transferred all the samples at the laboratory for quantification. The samples were analyzed in the laboratory of the Louvain centre for Toxicology and Applied Pharmacology (Université catholique de Louvain, Belgium) without knowledge of their exact provenance in a random sequence (blind analysis). The laboratory quantified blood cadmium and blood lead by Graphite Furnace Atomic Absorption Spectrometry (GFAAS); cadmium, chromium, cobalt, and nickel, in urine by means of inductively coupled argon plasma mass spectrometry (ICP-MS) on an Agilent 7500ce instrument; and urinary retinol binding protein (RBP) and albumin by latex immunoassay. Using the validated methods (GF-AAS and ICP-MS), the laboratory has obtained successful results in external quality assessment schemes organized by the Institute for Occupational, Environmental and Social Medicine of the University of Erlangen, Germany (G-EQUAS program), and by the Institut National de Santé Publique, Québec (PCI and QMEQAS programs). Except for albumin, urinary parameters were expressed in μg/g creatinine. We excluded from statistical analyses the urinary samples with creatinine concentrations < 0.3 g/l or > 3 g/l (10 children and 14 adults), because they represent abnormal dilution.

#### Questionnaire

We asked to participants to fill in a questionnaire, adapted differently for adults or children. The questionnaire consisted in five parts with information on: i) general and socio-demographical aspects, ii) housing and surroundings, iii) lifestyle and behavior, iv) health status, and v) food consumption habits. We used food frequency questions to estimate consumption of different categories of food, with a special emphasis on consumption of locally grown vegetables. The questionnaire for children aged four years or more included in addition a specific section with a brief behavioral screening called ‘Strengths and Difficulties Questionnaire’ (SDQ), which included 25 items (difficulties), divided into five categories: emotional symptoms, conduct problems, hyperactivity/inattention, peer relationship problems, and prosocial behavior [14]. A total score of difficulties ≤15 (over the 25 items) was considered as normal.

### Statistical methods

We encoded data with the software EPIDATA 3.0. We did statistical analyses using the software SPSS 16.0, SPSS 18.0, and STATA 10.1. We used a *p*-value ≤ 0.05 as level of significance. When needed, we performed log normal transformation. Based on the analysis of scatter and box plots, we excluded from analyses the abnormally extreme values (outliers). For urinary parameters, the concentrations under the limit of quantification (LOQ) received a value of 1/2 LOQ.

We compared, by univariate analysis, the concentrations of biomarkers between the central and peripheral areas, by age group and, in adults, by sex. For these univariate analysis, we used the Student’s t-test and analysis of variance (ANOVA) for mean comparisons. We used the Pearson’s correlation coefficient to test association between quantitative variables, unless normality of distribution was not reached, in case of which we used the Spearman’s correlation coefficient. We compared proportions between groups by the Chi-square test or the exact Fisher’s test.

We analyzed the determinants of each biomarker (metals and biomarkers of effects) by multivariate analysis. We applied stepwise multiple linear regressions with biomarker concentrations as dependent exposure variables on the combined groups of central and peripheral areas, for adults, children aged 7 to 11 years, and children aged 2.5 to 6 years separately. We tested the following independent variables (predictors) in the different models: age, sex (women = 0; men = 1), body mass index, geographical area (center = 0; periphery = 1), housing age (before 1950 = 0; 1950 and after = 1), lead pipes (presence = 1; absence = 0), consumption of locally grown vegetables (consumers = 1; others = 0), education (high school = 1; others = 0), smoking status (current smokers = 1; others = 0), location of work (municipality of Ath = 1; outside = 0), location of school (municipality of Ath = 1; outside = 0), alcohol consumption (daily consumption = 1; others = 0), having a prosthesis, treatment of anemia, vitamin B complex supplementation, and ferritin.

Based on the results of the multiple regression analyses, we adjusted metal concentrations for their significant determinants. We then assessed potential associations with lifestyle-related variables, in particular the consumption of locally grown vegetables.

## Results

### Characteristics of the study population

A total of 278 participants took part in this study. The study population consisted of 98 young children aged 2.5 to 6 years, 74 children aged 7 to 11 years and 106 adults (52 men and 54 women). The response rate reached 24 %. The most relevant characteristics of the study population are given in Table 1. There is no significant difference in mean age between central and peripheral areas, neither in children nor in adults. The most obvious differences between the central and peripheral areas related to the location of school, the location of work, and the locally grown vegetables consumption, which are all linked to the intrinsic characteristics of these two geographical groups. The study population had a high level of education, both in the central and the peripheral area.

**Table 1:** Baseline characteristics among study participants (*N* = 278) by age group and by area, Ath, Belgium, 2009

	Central area	Peripheral area	Total
Children aged 2.5 to 6 years	*n* = 49	*n* = 49	*n* = 98
Age, years^a^	4.6 (1.4)	4.5 (1.5)	4.6 (1.5)
Sex, *n*			
Male	27 (55 %)	24 (49 %)	51 (52 %)
Female	22 (45 %)	25 (51 %)	47 (48 %)
BMI, kg/m^2^ ^a^	16.0 (2.1)	16.1 (1.2)	16.0 (1.7)
Higher education of parents, *n*	43 (88 %)	42 (85 %)	85 (88 %)
School in the city of Ath, *n*	36 (78 %)	6 (12 %)	42 (44 %)
Locally grown vegetables consumption, *n*	17 (35 %)	33 (67 %)	50 (51 %)
Children aged 7 to 11 years	*n* = 38	*n* = 36	*n* = 74
Age, years^a^	9.4 (1.5)	9.6 (1.5)	9.5 (1.5)
Sex, *n*			
Male	22 (58 %)	20 (56 %)	42 (57 %)
Female	16 (42 %)	16 (44 %)	32 (43 %)
BMI, kg/m^2^ ^a^	17.8 (3.5)	17.4 (2.4)	17.6 (3.0)
Higher education of parents, *n*	28 (78 %)	28 (80 %)	56 (79 %)
School in the city of Ath, *n*	27 (77 %)	6 (18 %)	33 (48 %)
Locally grown vegetables consumption, *n*	19 (50 %)	25 (69 %)	44 (60 %)
Men (40–60 years)	*n* = 27	*n* = 25	*n* = 52
Age, years^a^	52.8 (5.7)	50.3 (6.2)	51.6 (6.0)
BMI, kg/m^2^ ^a^	30.9 (3.0)	28.0 (3.4)	31.1 (2.2)
Higher education, *n*	14 (52 %)	13 (54 %)	27 (53 %)
Work in Ath, *n*	10 (37 %)	6 (25 %)	16 (31 %)
Locally grown vegetables consumption, *n*	14 (52 %)	11 (44 %)	25 (48 %)
Daily consumption of alcohol, *n*	9 (35 %)	11 (44 %)	20 (39 %)
Current smokers, *n*	5 (19 %)	5 (20 %)	10 (20 %)
Women (40–60 years)	*n* = 28	*n* = 26	*n* = 54
Age, years^a^	50.3 (5.7)	51.3 (6.3)	50.8 (6.0)
BMI, kg/m^2^ ^a^	28.1 (5.7)	27.4 (7.1)	27.8 (6.3)
Higher education, *n*	21 (75 %)	16 (62 %)	37 (69 %)
Work in Ath, *n*	16 (59 %)	9 (39 %)	25 (50 %)
Locally grown vegetables consumption, *n*	11 (39 %)	20 (77 %)	31 (57 %)
Daily consumption of alcohol, *n*	7 (25 %)	9 (35 %)	16 (30 %)
Current smokers, *n*	6 (21 %)	4 (15 %)	10 (19 %)

^a^Arithmetic means (standard deviation). *BMI* body mass index

### Biomarkers of exposure

Table 2 shows the values of the biomarker concentrations in the central and peripheral areas, by age group and, in adults, by sex. We excluded as outliers six unusually high values. In children, the excluded values were one of nickel (23.6 μg/g cr), one of cobalt (2.16 μg/g cr), and one of chromium (1.04 μg/g cr). All three belonged to three different children of the peripheral area, with no significantly higher values for the other biomarkers. In adults, the excluded values were 23.2 μg chromium /g cr, 14.7 μg nickel /g cr, and 13.8 μg nickel /g cr. The two first belonged to a man of the peripheral area and might be related to his professional activity. The last one belonged to a woman of the central area and might be related to her leisure activities. The mean blood lead concentration of children aged 2.5 to 6 years was significantly higher in the central area than in the peripheral area. This is the only significant difference in mean of metals observed between the two geographical areas, in the three groups of age.

**Table 2:** Biomarker levels among study participants (*N* = 278) by age group and study area, Ath, Belgium, 2009

	Central area	Peripheral area	Total
Children (2.5 to 6 years)	*n* = 49	*n* = 49	*n* = 98
Blood lead, μg/l	18.2 (15.9–20.9) [6–66]	14.8 (12.6–17.4) [3–68]^a^	16.6 (14.8–18.2) [3–68]
Children (7 to 11 years)	*n* = 38	*n* = 36	*n* = 74
Blood lead, μg/l	15.5 (13.2–18.2)	14.1 (11.8–17.4) [4–50]	14.8 (13.2–16.6) [4–50]
Blood cadmium, μg/l	0.13 (0.11–0.15) [0–0.3]	0.14 (0.12–0.16) [0–0.2]	0.13 (0.12–0.15) [0–0.3]
Urinary cadmium, μg/g cr	0.07 (0.05–0.09) [0.01–0.22]	0.06 (0.04–0.07) [0.02–0.20]	0.06 (0.05–0.07) [0.01–0.22]
Urinary cobalt, μg/g cr	0.38 (0.29–0.50) [0.08–1.27]	0.29 (0.23–0.36) [0.09–0.68]	0.33 (0.28–0.39) [0.08–1.27]
Urinary chromium, μg/g cr	0.18 (0.15–0.21) [0.06–0.47]	0.18 (0.15–0.23) [0.07–0.48]	0.18 (0.16–0.21) [0.06–0.48]
Urinary nickel, μg/g cr	1.75 (1.46–2.09) [0.37–3.92]	1.75 (1.37–2.26) [0.30–5.74]	1.75 (1.51–2.03) [0.30–5.74]
Urinary RBP, μg/g cr	105.1 (86.5–128.7) [25.6–352]	98.2 (72.0–128.7) [17.1–735]	100.6 (85.0–119.2) [17.1–735]
Men (40–60 years)	*n* = 27	*n* = 25	*n* = 52
Blood lead, μg/l	31.2 (26.4–36.9) [14–85]	32.3 (26.1–40.0) [12–118]	31.7 (27.9–36.1) [12–118]
Blood cadmium, μg/l	0.25 (0.19–0.33) [0.1–1.2]	0.38 (0.29–0.50) [0.1–1.7]	0.30 (0.25–0.37) [0.1–1.7]
Urinary cadmium, μg/g cr	0.22 (0.16–0.30) [0.05–0.87]	0.20 (0.14–0.30) [0.04–0.78]	0.21 (0.17–0.27) [0.04–0.87]
Urinary cobalt, μg/g cr	0.15 (0.11–0.20) [0.06–0.58]	0.18 (0.13–0.25) [0.06–1.84]	0.16 (0.13–0.20) [0.06–1.84]
Urinary chromium, μg/g cr	0.13 (0.10–0.16) [0.05–0.33]	0.16 (0.13–0.21) [0.05–0.35]	0.14 (0.12–0.17) [0.05–0.35]
Urinary nickel, μg/g cr	0.65 (0.51–0.84) [0.21–2.58]	0.69 (0.51–0.92) [0.20–2.16]	0.67 (0.56–0.80) [0.20–2.58]
Urinary RBP, μg/g cr	96.0 (66.7–138.2) [2.9–388]	93.4 (79.9–109.8) [44.2–192]	94.9 (78.1–115.2) [2.9–388]
Urinary albumin, mg/l	7.85 (5.85–10.54) [5.6–52.4]	9.29 (6.40–13.49) [5.6–101]	8.52 (6.78–10.72) [5.6–101]
Women (40–60 years)	*n* = 28	*n* = 26	*n* = 54
Blood lead, μg/l	22.5 (18.0–28.2) [4–57]	20.3 (15.6–26.5) [6–93]	21.4 (18.1–25.3) [4–93]
Blood cadmium, μg/l	0.37 (0.29–0.47) [0.2–1.9]	0.39 (0.32–0.47) [0.2–1.6]	0.38 (0.33–0.44) [0.2–1.9]
Urinary cadmium, μg/g cr	0.23 (0.17–0.33) [0.04–0.78]	0.25 (0.18–0.34) [0.05–1.0]	0.25 (0.20–0.30) [0.04–1.0]
Urinary cobalt, μg/g cr	0.20 (0.16–0.26) [0.07–0.40]	0.22 (0.17–0.29) [0.09–1.12]	0.21 (0.18–0.25) [0.07–1.12]
Urinary chromium, μg/g cr	0.17 (0.14–0.20) [0.06–0.32]	0.18 (0.15–0.21) [0.07–0.29]	0.17 (0.15–0.20) [0.06–0.32]
Urinary nickel, μg/g cr	1.30 (1.05–1.61) [0.66–3.25]	0.96 (0.72–1.29) [0.17–2.02]	1.10 (0.91–1.33) [0.17–3.25]
Urinary RBP, μg/g cr	121.4 (97.9–159.4) [47.1–411]	108.5 (87.5–134.5) [38.2–270]	115.9 (99.1–135.5) [38.2–411]
Urinary albumin, mg/l	11.05 (6.94–17.60) [5.6–179]	8.44 (6.15–11.60) [5.6–46.7]	9.57 (7.33–12.50) [5.6–179]

Data are geometric means (95 % Confidence Interval) [minimum-maximum]. ^a^Statistically significant difference (*p* <0.05) between central and peripheral area using the Student’s t-test, *cr* creatinine, *RBP* retinol-binding protein

In children aged 7 to 11 years, the mean blood lead and urinary cobalt concentrations were higher in the central area than in the peripheral area, but without reaching the level of significance. For cobalt in children, we observed a higher range in the central area, with a maximum value being almost twice the maximum value observed in the peripheral area. In addition, 25 % of cobalt values in children from the central area exceeded the maximum value observed in the peripheral area.

### Biomarkers of effect

We analyzed retinol-binding protein (RBP) and microalbuminuria as biomarkers of effect, in order to investigate potential health damages induced by heavy metals. We observed no significant mean difference in the studied parameters between central and peripheral areas, neither for children or adults (Table 2).

For urinary albumin, despite the high number of values exceeding the microalbuminuria threshold of 20 mg/l (19 % of values in men and 22 % in women), we saw no correlation with cadmium concentrations.

For RBP, five values (three in children and two in adults) exceeded the threshold value of 300 μg/g creatinine. However, these values were not associated with high cadmium concentrations. In adults, we observed no correlation between RBP and cadmium values. In children, a significant positive correlation between RBP and urinary cadmium values appeared (*r*^2^ = 0.24; *p* <0.0001).

### Determinants of biomarker concentrations

We performed multiple regression analyses, stratified by age group, in order to identify determinants of biomarker concentrations. Table 3 shows the significant determinants identified by the models. The only parameter for which the geographical area was a significant determinant was blood lead in children aged 2.5 to 6 years. For this age group, no other determinant were found significant. In children aged 7 to 11 years, blood lead concentrations were significantly influenced by the housing age, children living in houses built before 1950 having higher concentrations. We found no significant determinant for cobalt, chromium or nickel in this age group. Blood lead concentrations in adults were not only influenced by sex (men having higher values) but also positively related with alcohol consumption and age, and negatively related with BMI. In adults, the main determinant of blood cadmium was smoking status (explaining 30 % of the variation of concentrations). Urinary cadmium, however, more closely related to chronic exposure, was mainly influenced by age (explaining 20 % of the variation of concentrations). Regarding biomarkers of effect, urinary RBP was highly determined in children by urinary cadmium, the only significant determinant for this parameter. In adults, we found no determinant, neither for RBP nor for microalbuminuria.

**Table 3:** Multiple linear regression analyses: significant determinants of biomarkers among study participants by age group, Ath, Belgium, 2009

Dependent variable	Independent variable	Partial r^2^	β estimate	*p*-value
Children aged 2.5 to 6 years				
Blood lead, μg/l	Geographical area	0.40	−0.092	0.05
Children aged 7 to 11 years				
Blood lead, μg/l	Housing age	0.129	−0.141	0.005
	BMI	0.081	−0.020	0.016
Urinary cadmium, μg/g cr	BMI	0.159	−0.038	0.002
Urinary RBP, μg/g cr	Urinary cadmium	0.241	0.491	<0.001
Adults (40-60 years)				
Blood lead, μg/l	Sex	0.115	−0.158	0.001
	Alcohol consumption	0.072	0.010	0.027
	Age	0.057	0.010	0.020
	BMI	0.038	−0.010	0.033
Blood cadmium, μg/l	Smoking status	0.304	0.388	0.001
	Ferritine	0.046	−0.120	0.008
	Age	0.030	0.011	0.005
Urinary cadmium, μg/g cr	Age	0.211	0.022	0.001
	Smoking status	0.056	0.219	0.016

*cr* creatinine, *BMI* body mass index, *RBP* retinol-binding protein

### Analyses on adjusted values

In order to take into account the effect of age, smoking status, etc, we adjusted metal concentrations for the significant variables identified by the multiple regression analyses. We then further assessed potential associations between adjusted metal concentrations and various determinants, like the consumption of locally grown vegetables. We obtained the locally grown vegetables consumption data from the questionnaire. We defined it as the proportion of the total annual consumption of vegetables coming from the garden of the participant or from a vegetable garden of the neighborhood. We tested mean differences of adjusted metal values between categories of consumption of locally grown vegetables (no consumption; <50 % of the total consumption; and ≥50 % of the total consumption) separately in central and peripheral areas. The proportion of adults consuming locally grown vegetables was 53 %. We observed no significant difference of heavy metal levels in the body of participants according to consumption categories, whatever the metal, category of age, or geographical area.

We assessed other lifestyle-related determinants of blood lead levels in children aged 2.5 to 6 years by area, according to the information from the questionnaire. We observed no effect of sucking one's thumb, biting one's nails, or of the frequency of hand washing. On the other hand, time spent outdoors turned out to be a significant determinant of blood lead levels in the central area, but not in the peripheral area. In the central area, children aged 2.5 to 6 years spending more than 5 h per week outdoors had significantly higher blood lead levels than those spending 5 h or less per week outdoors (24.8 μg/l vs. 16.5 μg/l respectively, *p* = 0.009). Moreover, the same effect of time spent outdoors was seen in children aged 7 to 11 years in the central area (19.4 μg/l when >5 h/week vs. 13.18 μg/l when ≤5 h/week, *p* = 0.015) but not in the peripheral area.

Finally, we assessed the association between blood lead concentrations and the behavioral score obtained by the Strengths and Difficulties Questionnaire (SDQ). We obtained the behavioral scores for 143 children aged ≥4 years. We observed no correlation between the total score of difficulties from the SDQ and the blood lead levels. We observed neither significant geometric mean difference in blood lead between children with normal scores (≤15; *n* = 132) and children with higher scores (>15; *n* = 11).

## Discussion

The only significant difference between the central area, where the two plants are located, and the peripheral area, was the higher blood lead levels among children aged 2.5 to 6 years living in the central area. The other metals, as well as the biomarkers of effects, and the lead concentrations in adults and in children aged 7 to 11 years, did not differ between the two areas.

### Biomarkers of exposure

The metal concentrations observed in this study, in a population living in the surroundings of two non-ferrous metal plants, were overall in the same range to what was found in the general population in Belgium [15–18] or other industrialized countries such as France [19]; Germany [20–23]; other European countries [24]; United States [25, 26]; or Canada [27, 28]. The transfer of the environmental pollution to residents in the vicinity of the plants in Ath seems therefore to be limited. And the expected health outcomes in the residents are then negligible. The blood lead and urinary cadmium concentrations observed in our study were clearly lower than what has been found in previous studies [11, 29–32] performed in the general population several years or decades ago, among which the Cadmibel study [32], on populations environmentally exposed to cadmium by non-ferrous smelters in Belgium. This reflected a global decrease in exposure that has been occurring over several decades.

### Biomarkers of effects

RBP and albumin are proteins whose excretion increases in case of cadmium-induced renal damage [11, 33]. Given the relatively low cadmium levels observed in our study, high levels of these biomarkers of effects were not expected and, if any, could hardly be related to metals exposure. The high number of values exceeding the microalbuminuria threshold observed in this study could then more likely be due to other known factors affecting urine albumin excretion such as diabetes, hypertension, or intensive exercise. Regarding RBP, the health implication of the statistically significant relationship observed at low environmental cadmium exposures and with low RBP levels is difficult to assess. Recent studies showed that association of proteinuria with low-level urinary cadmium can be largely driven by variations in diuresis and probably also the co-excretion of cadmium with proteins [34–36]. Urinary cadmium at very low exposure levels would maybe no longer be a reflection of the body burden but would be significantly influenced by the elimination of cadmium by the kidneys and in particular the co-excretion of the metal with urinary proteins. The parallel increase in urinary cadmium and RBP could then reflect the physiological variation in the tubular reabsorption capacity of the proximal tubule, rather than kidney damage caused by cadmium.

### Children versus adults

While heavy metal concentrations in adults were similar between central and peripheral areas, some differences appeared in children that might be seen as the consequence of the pollution from the plants. In the central area, the higher lead concentrations seen in children but not in adults could be due to the higher gastro-intestinal absorption of lead in children (40–50 %) than in adults (3–10 %). Moreover, the time spent outdoors was significantly associated with higher blood lead levels in children living at proximity of the plants. This could result from specific behavior of children, in particular hand-to-mouth activity. Nevertheless, all the blood lead values observed in children in Ath were below the value of 100 μg/l, which is the level of concern used until recently [37, 38]. Among studied children, three had blood lead values exceeding the more stringent threshold of 50 μg/l. But even this threshold cannot ensure absence of toxicity, as no safe lead-exposure threshold can be identified to date and more and more studies report health effects at low blood lead levels. Low-level exposure to lead during early childhood is associated with lower neuropsychological development through the first 7 years of live, with delayed puberty, and with a lower intelligence quotient (IQ) [3, 39–41]. Moreover, the steepest declines in IQ occur at blood levels <100 μg/l [5]. Nevertheless, in our study, we have not observed association between blood lead concentrations and behavioral score. We cannot exclude that this was a consequence of the limited number of observations. Given the recent findings about health effects at low blood lead levels, the German Human Biomonitoring Commission suspended their HBM value of 100 μg/l for lead in blood and set a reference value of 35 μg/l [42, 43]. In the United States, the Center for Disease Control and Prevention (CDC) also recommended to replace the blood lead “level of concern” of 100 μg/l and recommended the use of 50 μg/l as new reference value [44].

While, for cumulative metals such as cadmium and lead, concentrations observed in children were, as expected, lower than those observed in adults, the concentrations of cobalt, chromium and nickel, which reflect recent exposure, were similar or even higher in children than in adults. These differences could result from differences in exposures, but also in physiology. Moreover, the safety factors concerning health effects may differ between adults and children. Therefore, the adult reference values have to be used with caution for children. In Germany, specific reference values for children have been established. For example, the urinary nickel reference value for children is higher than the one for adults, and corresponds to 4.5 μg/l [22]. In Belgium, an update of references values was recently established for adults [16], and the FLEHS study gave representative mean values for a large number of pollutants in newborns, adolescents, and adults in Flanders [15].

### Public health relevance

This study was requested by local and regional authorities because of their and the population concern, about the potential health risks of living close to two non-ferrous metal plants still operating. The results of the study indicated that the health effects associated to living in the vicinity of the plants were expected to be negligible in the studied population. The study was used as a tool to assess the right action to undertake in terms of public health facing the presence of the plants. Given the results of this study, we recommended no specific action. However, considering the absence of a known threshold for lead toxicity, a particular attention at individual level to limit as much as possible all ways of lead exposure, in particular for children, could be of benefit for the population of Ath in the same way that for the general population. From a general point of view, we think that this kind of very targeted HBM study is a good tool to help authorities to assess public health impact of specific situations such as hot-spots of pollution. The objectives of this kind of local HBM are however different from those of large scale HBM. These last ones are developed to identify priority exposure, to establish reference ranges for comparison, to study time trends, or to evaluate exposure prevention efforts, generally at a national or supra-national scale. Whereas in the case of hot-spot studies, the study population is of limited size but has the advantage to be well defined and to be representative of the local population.

### Strengths and limitations

Among the strengths of this study are the random recruitment of study population by a professional survey firm; data collection at a single site, in a short period of time, with harmonized protocols, by well-trained staff; and the study population including not only adults but also children of different age groups. Several limitations of this study are also to be considered. First, the limited number of participants, corresponding to the expected sample size to detect a difference of 30 % in comparison to representative blood lead value. This choice was a balanced compromise between statistical power and financial constraints. This percentage can be compared to the variations observed in the general population. For example, the mean urinary cadmium of women in the different European countries that participated to the DEMOCOPHES study vary between 0.118 μg/g creatinine and 0.379 μg/g creatinine [45]. This represent respectively 35.5 % less and 105.4 % more than the Belgian average of 0.183 μg/g creatinine [45]. Moreover, we expected no public health consequences with an increase of 30 % of the observed values, as the 95 percentiles would remain below the respective references values. However, we cannot exclude that with a higher sample size, some small differences could have reach the level of significance. A second limitation of this study is the response rate that reached only 24 %. Response rates vary a lot between the different HBM studies. In the GerES, where the HBM was coupled to the National Health Interview and Examination Surveys (NHIES), the response rate was up to 73 % [46]. But other HBM studies reported response rates comparable to our study (e.g. the Flemish Human Biomonitoring: 22.3 %). The recruitment method is a parameter that could have influenced the response rate. Another is the importance of the requested contribution (displacement for sampling, blood and urine sample collection, questionnaire). Nevertheless, the obtained response rate does not exclude a risk of selection bias. Finally, another potential selection bias could limit our study. Individuals of lower socio-economic status (SES) are less likely to participate in health surveys [47]. In our study, the educational level, used as a proxy for the SES, is higher than in the general population. Therefore, we cannot exclude that we missed part of population with lower SES, due to non-participation. As a lower SES is generally associated with higher exposure to metals (lead, nickel) [20, 48], the possible selection bias would therefore lead to underestimation of exposure. This bias would however affect both the central and the peripheral areas.

## Conclusions

The comparison of human exposure to heavy metals in Ath, between a central area where two non-ferrous metal plants are located, and a peripheral area, shows only slightly higher blood lead levels among young children in the vicinity of the plants. Despite the presence of the two plants, the concentrations of heavy metals found in the population of Ath are comparable to those found in other similar biomonitoring studies performed among the general population and remain below the levels of concern for public health. We therefore recommended no specific action to follow the population living close to these plants.

## Abbreviations

BMI: Body mass index
HBM: Human BioMonitoring
IQ: Intelligence quotient
LOQ: Limit of quantification
RBP: Retinol-binding protein
SES: Socio-economic status

## References

[1] Jarup L (2003). Hazards of heavy metal contamination. Br Med Bull.

[2] de Burbure C, Buchet JP, Leroyer A, Nisse C, Haguenoer JM, Mutti A (2006). Renal and neurologic effects of cadmium, lead, mercury, and arsenic in children: evidence of early effects and multiple interactions at environmental exposure levels. Environ Health Perspect.

[3] U.S. National Toxicology Program. NTP Monograph on Health Effects of Low-Level Lead. Washington, DC: National Institutes of Health (NIH); 2012. Publication No. 12-5996, ISSN 2330-1279. 23964424

[4] Spivey A (2007). The weight of lead. Effects add up in adults. Environ Health Perspect.

[5] Jakubowski M (2011). Low-level environmental lead exposure and intellectual impairment in children--the current concepts of risk assessment. Int J Occup Med Environ Health.

[6] Leroyer A, Nisse C, Hemon D, Gruchociak A, Salomez JL, Haguenoer JM (2000). Environmental lead exposure in a population of children in northern France: factors affecting lead burden. Am J Ind Med.

[7] Bernard A (2008). Cadmium & its adverse effects on human health. Indian J Med Res.

[8] Institut Provincial d’Hygiène et de bactériologie du Hainaut (IPHB). Vigilance Sanitaire de la Ville d’Ath, Etude environnementale. Rapport; 2007.

[9] DGRNE. Réseau de mesure de la qualité de l’air en Région wallonne. Rapport; 2004.

[10] Rebolledo J, Fierens S, Versporten A, Brits E, De Plaen P, Van Nieuwenhuyse A (2011). Human biomonitoring on heavy metals in Ath: methodological aspects. Arch Public Health.

[11] Buchet JP, Lauwerys R, Roels H, Bernard A, Bruaux P, Claeys F (1990). Renal effects of cadmium body burden of the general population. Lancet.

[12] de Jong PE, Curhan GC (2006). Screening, monitoring, and treatment of albuminuria: Public health perspectives. J Am Soc Nephrol.

[13] Berglund M, Akesson A, Nermell B, Vahter M (1994). Intestinal absorption of dietary cadmium in women depends on body iron stores and fiber intake. Environ Health Perspect.

[14] Goodman R (1997). The Strengths and Difficulties Questionnaire: a research note. J Child Psychol Psychiatry.

[15] Baeyens W, Vrijens J, Gao Y, Croes K, Schoeters G, Den Hond E (2014). Trace metals in blood and urine of newborn/mother pairs, adolescents and adults of the Flemish population (2007–2011). Int J Hyg Environ Health.

[16] Hoet P, Jacquerye C, Deumer G, Lison D, Haufroid V (2013). Reference values and upper reference limits for 26 trace elements in the urine of adults living in Belgium. Clin Chem Lab Med.

[17] Pirard C, Koppen G, De Cremer K, Van Overmeire I, Govarts E, Dewolf MC (2014). Hair mercury and urinary cadmium levels in Belgian children and their mothers within the framework of the COPHES/DEMOCOPHES projects. Sci Total Environ.

[18] Schroijen C, Baeyens W, Schoeters G, Den Hond E, Koppen G, Bruckers L (2008). Internal exposure to pollutants measured in blood and urine of Flemish adolescents in function of area of residence. Chemosphere.

[19] Etchevers A, Bretin P, Lecoffre C, Bidondo ML, Le Strat Y, Glorennec P (2014). Blood lead levels and risk factors in young children in France, 2008–2009. Int J Hyg Environ Health.

[20] Becker K, Müssig-Zufika M, Conrad A, Lüdecke C, Schulz C, Seiwert M, Kolossa-Gehring M. German Environmental Survey for Children 2003/06 - GerES IV - Human Biomonitoring: Levels of selected substances in blood and urine of children in Germany. Federal Environment Agency (Umweltbundesamt); 2008.

[21] Kolossa-Gehring M, Becker K, Conrad A, Ludecke A, Riedel S, Seiwert M (2007). German Environmental Survey for Children (GerES IV)--first results. Int J Hyg Environ Health.

[22] Schulz C, Angerer J, Ewers U, Heudorf U, Wilhelm M (2009). Revised and new reference values for environmental pollutants in urine or blood of children in Germany derived from the German environmental survey on children 2003–2006 (GerES IV). Int J Hyg Environ Health.

[23] Wilhelm M, Schulz C, Schwenk M (2006). Revised and new reference values for arsenic, cadmium, lead, and mercury in blood or urine of children: basis for validation of human biomonitoring data in environmental medicine. Int J Hyg Environ Health.

[24] Den Hond E, Govarts E, Willems H, Smolders R, Casteleyn L, Kolossa-Gehring M (2015). First steps toward harmonized human biomonitoring in Europe: demonstration project to perform human biomonitoring on a European scale. Environ Health Perspect.

[25] Calafat AM (2012). The U.S. National Health and Nutrition Examination Survey and human exposure to environmental chemicals. Int J Hyg Environ Health.

[26] U.S. Centers for Disease Control and Prevention (CDC). Fourth National Report on Human Exposure to Environmental Chemicals. Update Tables. Atlanta, GA; U.S. Department of Health and Human Services, Centers for Disease Control and Prevention; 2014. http://www.cdc.gov/exposurereport/.

[27] Wong S, Lye E. Enquête canadienne sur les mesures de la santé: Taux de plomb, de mercure et de cadmium chez les Canadiens. 2008. Report No.: 19 (4).

[28] Health Canada. Second report on human biomonitoring of environmental chemicals in Canada. Ottawa: Canadian Federal Department of Health (Health Canada); 2013.

[29] Becker K, Schroeter-Kermani C, Seiwert M, Ruther M, Conrad A, Schulz C (2013). German health-related environmental monitoring: assessing time trends of the general population’s exposure to heavy metals. Int J Hyg Environ Health.

[30] Bretin P, Garnier R, Chatelot J, Lecoffre C, Delour M, Cheymol J (2008). Childhood lead poisoning screening in France since 1995: practices, results, trends and recommendations. Bulletin Epidemiol Hebd.

[31] Fierens S, Mairesse H, Heilier JF, Focant JF, Eppe G, De Pauw E (2007). Impact of iron and steel industry and waste incinerators on human exposure to dioxins, PCBs, and heavy metals: results of a cross-sectional study in Belgium. J Toxicol Environ Health A.

[32] Hotz P, Buchet JP, Bernard A, Lison D, Lauwerys R (1999). Renal effects of low-level environmental cadmium exposure: 5-year follow-up of a subcohort from the Cadmibel study. Lancet.

[33] Nordberg G, Jin T, Wu X, Lu J, Chen L, Liang Y (2012). Kidney dysfunction and cadmium exposure--factors influencing dose-response relationships. J Trace Elem Med Biol.

[34] Chaumont A, Nickmilder M, Dumont X, Lundh T, Skerfving S, Bernard A (2012). Associations between proteins and heavy metals in urine at low environmental exposures: evidence of reverse causality. Toxicol Lett.

[35] Chaumont A, Voisin C, Deumer G, Haufroid V, Annesi-Maesano I, Roels H (2013). Associations of urinary cadmium with age and urinary proteins: further evidence of physiological variations unrelated to metal accumulation and toxicity. Environ Health Perspect.

[36] Haddam N, Samira S, Dumont X, Taleb A, Lison D, Haufroid V (2011). Confounders in the assessment of the renal effects associated with low-level urinary cadmium: an analysis in industrial workers. Environ Health.

[37] Centers for Desease Control and Prevention (CDC). Preventing lead poisoning in young children. Atlanta, GA: U.S. Department of Health and Human Services, Centers for Disease Control and Prevention; 1991.

[38] International Programme on Chemical Safety. Inorganic lead. Environmental health criteria 165. World Health Organisation, editor. Geneva: World Health Organisation (WHO); 1995.

[39] Canfield RL, Henderson CR, Cory-Slechta DA, Cox C, Jusko TA, Lanphear BP (2003). Intellectual impairment in children with blood lead concentrations below 10 microg per deciliter. N Engl J Med.

[40] Sanders T, Liu Y, Buchner V, Tchounwou PB (2009). Neurotoxic effects and biomarkers of lead exposure: a review. Rev Environ Health.

[41] Sioen I, Den Hond E, Nelen V, Van De Mieroop E, Croes K, Van Larebeke N (2013). Prenatal exposure to environmental contaminants and behavioural problems at age 7-8years. Environ Int.

[42] Schulz C, Wilhelm M, Heudorf U, Kolossa-Gehring M (2011). Update of the reference and HBM values derived by the German Human Biomonitoring Commission. Int J Hyg Environ Health.

[43] Wilhelm M, Heinzow B, Angerer J, Schulz C (2010). Reassessment of critical lead effects by the German Human Biomonitoring Commission results in suspension of the human biomonitoring values (HBM I and HBM II) for lead in blood of children and adults. Int J Hyg Environ Health.

[44] Centers for Desease Control and Prevention (CDC) Advisory Committee on Childhood Lead Poisoning Prevention. Low level lead exposure harms children: a renewed call for primary prevention. Atlanta, GA: U.S. Department of Health and Human Services, Centers for Disease Control and Prevention; 2012.

[45] Smolders R, Den Hond E, Koppen G, Govarts E, Willems H, Casteleyn L (2015). Interpreting biomarker data from the COPHES/DEMOCOPHES twin projects: Using external exposure data to understand biomarker differences among countries. Environ Res.

[46] Schulz C, Conrad A, Becker K, Kolossa-Gehring M, Seiwert M, Seifert B (2007). Twenty years of the German Environmental Survey (GerES): human biomonitoring--temporal and spatial (West Germany/East Germany) differences in population exposure. Int J Hyg Environ Health.

[47] Lorant V, Demarest S, Miermans PJ, Van Oyen H (2007). Survey error in measuring socio-economic risk factors of health status: a comparison of a survey and a census. Int J Epidemiol.

[48] Hu H, Shih R, Rothenberg S, Schwartz BS (2007). The epidemiology of lead toxicity in adults: measuring dose and consideration of other methodologic issues. Environ Health Perspect.

